# Association of Fetal Intrahepatic Umbilical–Portal Anastomosis Type with Late-Onset Fetal Growth Restriction and Neonatal Outcomes

**DOI:** 10.3390/diagnostics16142253

**Published:** 2026-07-19

**Authors:** Kubilay Çanga, Recep Taha Ağaoğlu, Özgür Volkan Akbulut, Mustafa Bağcı, Emel Özalp, Muhammed Alp Özdemir, Yüksel Oğuz, Kadriye Yakut Yücel

**Affiliations:** 1 Division of Perinatology, Department of Obstetrics and Gynecology, Etlik City Hospital, Ministry of Health, Ankara 06170, Türkiye; tahaagaoglu@hotmail.com (R.T.A.); akbulutvolkan@yahoo.com (Ö.V.A.); ykskoguz@hotmail.com (Y.O.); yakutkadriye@hotmail.com (K.Y.Y.); 2 Division of Perinatology, Department of Obstetrics and Gynecology, Sakarya Training and Research Hospital, Ministry of Health, Sakarya 54100, Türkiye; mustafabagci@outlook.com.tr; 3 Department of Obstetrics and Gynecology, Etlik City Hospital, Ministry of Health, Ankara 06170, Türkiye; emelozalp@gmail.com; 4 Department of Forensic Medicine, University of Health Sciences, Bursa Yuksek Ihtisas Training and Research Hospital, Bursa 16310, Türkiye; drozdemiralp@gmail.com

**Keywords:** fetal growth restriction, umbilical–portal anastomosis, fetal hepatic circulation

## Abstract

**Objective:** To evaluate whether fetal intrahepatic umbilical–portal anastomosis type is associated with late-onset fetal growth restriction (FGR) and adverse neonatal outcomes. **Methods:** This prospective, single-center case–control study included 160 singleton pregnancies in the third trimester: 80 with late-onset FGR defined according to Delphi consensus criteria and 80 appropriate-for-gestational-age (AGA) controls. Umbilical–portal anastomosis was assessed by ultrasound and classified as T-shaped, X-shaped, or H-shaped. Maternal, sonographic, obstetric, and neonatal data were recorded. Multivariable logistic regression evaluated associations with FGR, and Firth’s penalized regression assessed composite adverse perinatal outcome (CAPO) within the FGR group. **Results:** X-shaped anastomosis predominated in FGR (55.0%; T-shaped, 26.3%; H-shaped, 18.8%), whereas T-shaped was most common in controls (61.3%; X-shaped, 23.8%; H-shaped, 15.0%). FGR cases showed higher umbilical artery pulsatility index, lower middle cerebral artery pulsatility index and cerebroplacental ratio, earlier delivery, lower birth weight, and higher rates of NICU (neonatal intensive care unit) admission, transient tachypnea of the newborn, and CAPO (35% vs. 15%). Both X-shaped and H-shaped types were independently associated with FGR, with a stronger effect for X-shaped (adjusted odds ratio [aOR], 5.27 vs. 2.85). Predicted probabilities increased stepwise from T-shaped to H-shaped to X-shaped (30.3%, 55.3%, and 69.6%). Within the FGR group, only X-shaped anastomosis was independently associated with CAPO, whereas Doppler indices were not discriminatory. **Conclusions:** X-shaped fetal intrahepatic umbilical–portal anastomosis was independently associated with both late-onset FGR and adverse neonatal outcome. Intrahepatic vascular configuration may provide additional value beyond conventional fetal biometry and arterial Doppler in risk stratification.

## 1. Introduction

FGR, historically referred to as intrauterine growth restriction, describes a fetus that fails to achieve its genetically determined growth potential, most commonly due to placental dysfunction [1]. It remains a major contributor to stillbirth, neonatal morbidity, and long-term neurodevelopmental and cardiometabolic disease, making timely recognition a central objective of modern fetal medicine [2]. Current guidelines emphasize that assessment should extend beyond fetal size to include hemodynamic evaluation, focusing on whether the fetus is adapting to placental insufficiency [3].

Late-onset FGR, defined according to the Delphi consensus as fetal growth restriction diagnosed after 32 weeks of gestation, represents the form that most frequently challenges third-trimester obstetric surveillance [4]. Compared with early-onset disease, placental lesions are typically more subtle, umbilical artery Doppler findings may remain within normal limits, and clinically significant fetal compromise may develop despite only mild abnormalities on routine assessment [5]. These characteristics have driven ongoing interest in additional sonographic markers that more directly reflect fetal adaptive physiology rather than fetal size alone.

The fetal umbilical–portal venous system is of particular interest in this context, as it lies at the intersection of oxygen delivery, nutrient distribution, and hepatic perfusion [6]. The exact embryonic time at which T-shaped, X-shaped, and H-shaped configurations become fixed has not been directly established. The portal venous system is nevertheless formed by the end of the 10th gestational week after early remodeling of the paired vitelline and umbilical veins, supporting their interpretation as congenital configurations rather than shapes acquired later in gestation [6]. Prenatally, the configuration is demonstrated by the relationship between the main portal vein and portal sinus on two- or three-dimensional grayscale imaging with color or high-definition flow Doppler; reproducible assessment has been reported from 14 to 36 weeks of gestation [7]. Data on configuration-specific hemodynamics remain limited. A recent prospective study found lower umbilical venous perfusion and compensatory higher normalized main portal venous flow in FGR overall, but no significant flow differences among T-shaped, X-shaped, and H-shaped configurations [8]. Thus, any functional effect may relate to subtle regional hepatic flow distribution rather than a simple difference in total main portal venous flow.

Importantly, the role of the portal venous system in FGR extends beyond overt structural abnormalities. The fetal liver receives a substantial proportion of oxygenated placental blood, and the balance between hepatic perfusion and ductus venosus shunting represents a key component of fetal adaptation to hypoxemia and nutrient deprivation [9]. In growth-restricted fetuses, hepatic venous perfusion is reduced, and umbilical blood flow is preferentially redistributed toward the left hepatic lobe at the expense of the right, suggesting active remodeling of hepatic circulation under placental stress [10]. Consistently, studies of liver circulation in FGR have demonstrated reduced umbilical venous contribution to the right hepatic lobe, accompanied by compensatory redistribution through the portal and splanchnic circulation. Together, these findings indicate that the portal venous system is not merely a passive reflection of fetal compromise but an active component of the adaptive response, and that venous parameters may reveal impairment not fully captured by conventional arterial Doppler [11,12].

This physiological framework supports focusing on the umbilicoportal junction in late-onset FGR. T-shaped, X-shaped, and H-shaped anastomoses are not pathological shunts. However, they represent distinct vascular configurations at a key interface. This is where oxygenated placental blood is divided between hepatic perfusion and ductus venosus flow. In reduced placental inflow, these configurations may affect intrahepatic blood distribution. This may influence the fetus’s metabolic reserve during chronic placental insufficiency. Therefore, umbilical–portal anastomosis may not be a simple anatomical variant. It may function as a structural factor shaping fetal adaptive capacity.

Despite growing interest in the fetal venous circulation, an important knowledge gap remains. Existing studies have largely focused on major venous malformations, quantitative flow measurements, or small exploratory cohorts, whereas the prognostic significance of normal-range intrahepatic umbilical–portal anastomotic configurations has not been adequately defined, particularly in well-characterized late-onset FGR populations. The present study was therefore designed to evaluate the distribution of fetal umbilical–portal anastomosis types in pregnancies complicated by late-onset FGR and to investigate their association with neonatal outcomes.

## 2. Materials and Methods

This prospective, single-center observational case–control study was conducted between January 2025 and November 2025 at the Perinatology Clinic of Ankara Etlik City Hospital, a tertiary referral center. The study population consisted of pregnant women who were either referred to the perinatology outpatient clinic or hospitalized in the perinatology unit during the study period. Consecutive eligible singleton pregnancies that underwent third-trimester fetal ultrasound examination and delivered at the same center were screened. Pregnancies were classified into either the late-onset FGR group or the AGA control group according to predefined criteria. The FGR and AGA groups were matched for maternal age and pre-pregnancy body mass index (BMI). The final study cohort comprised 160 pregnancies, including 80 late-onset FGR and 80 AGA cases. The study protocol was approved by the Ethics Committee of Ankara Etlik City Hospital (14 February 2024; AEŞH BADEK-2024-135), and written informed consent was obtained from all participants prior to inclusion. 

Late-onset FGR was defined according to the Delphi consensus criteria for cases diagnosed after 32 + 0 weeks of gestation. Accordingly, a fetus was classified as late-onset FGR if the abdominal circumference (AC) or estimated fetal weight (EFW) was below the 3rd percentile, or if AC/EFW was below the 10th percentile in combination with indicators of placental dysfunction. Indicators of placental dysfunction included an umbilical artery pulsatility index (UA-PI) above the 95th percentile, a cerebroplacental ratio (CPR) below the 5th percentile, or a decline of more than two quartiles in serial biometric measurements [13].

Inclusion criteria were reliable gestational age determination, presence of a live singleton fetus, complete fetal biometry and Doppler assessment, successful sonographic visualization of the intrahepatic umbilical–portal venous system, and availability of complete delivery and neonatal outcome data. Gestational age was determined based on first-trimester crown–rump length (CRL) measurements. The control group consisted of fetuses with EFW between the 10th and 90th percentiles, normal amniotic fluid volume, and no sonographic evidence of placental insufficiency. To minimize confounding factors related to non-placental causes of growth restriction or neonatal morbidity, predefined exclusion criteria were applied. These included multiple gestation, uncertain gestational age, major fetal structural or genetic anomalies, congenital infections, persistent right umbilical vein, agenesis of the ductus venosus, significant umbilical–portal–systemic venous shunts or other major fetal venous malformations, inadequate visualization of the umbilicoportal junction, and major maternal systemic diseases such as chronic hypertension or preeclampsia, pregestational diabetes mellitus, renal disease, or autoimmune disorders.

**Figure 1:**
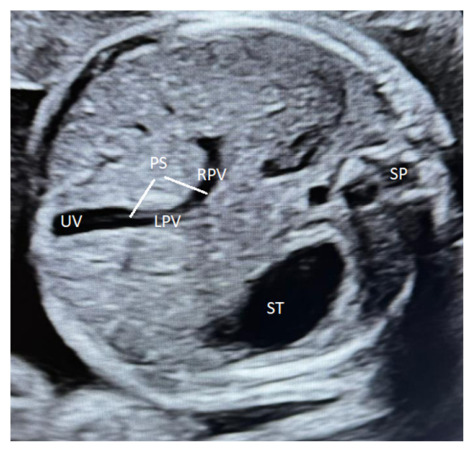
Normal intrahepatic umbilical–portal venous connection on transverse abdominal ultrasound. UV, umbilical vein; PS, portal sinus; RPV, right portal vein; LPV, left portal vein; SP, spine; ST, stomach.

Ultrasound examinations were performed in the third trimester using a Voluson S10 Expert ultrasound system (GE Healthcare, Milwaukee, WI, USA) equipped with a 3.5-MHz convex transducer. All scans were carried out transabdominally by a single experienced maternal–fetal medicine specialist (K.Ç.), thereby minimizing interobserver variability. Examinations were conducted in the standard transverse plane of the fetal upper abdomen used for abdominal circumference measurement, with a right-sided approach and the fetal stomach positioned away from the transducer. In this plane, the stomach and the L-shaped portal sinus (PS) were visualized, with the PS defined as the vascular space extending between the origin of the inferior branch of the left portal vein and that of the right portal vein (Figure 1). The junction between the PS and the main portal vein (MPV) was assessed by a rightward and downward sweep, using the hepatic artery adjacent to the MPV as a reference landmark. Evaluation of the fetal umbilical–portal venous system was complemented by a longitudinal plane to visualize the umbilical vein and the ductus venosus.

**Figure 2:**
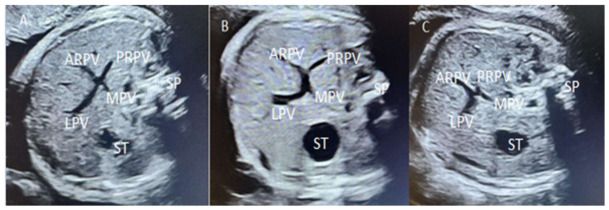
Types of fetal intrahepatic umbilical–portal venous anastomoses on transverse abdominal ultrasound. Panels correspond to (**A**) T-shaped, (**B**) X-shaped, and (**C**) H-shaped configurations. ARPV, anterior right portal vein; PRPV, posterior right portal vein; MPV, main portal vein; LPV, left portal vein; SP, spine; ST, stomach.

In this study, the type of umbilical–portal anastomosis was evaluated as T-shaped, X-shaped, or H-shaped according to the sonographic classification described by Kivilevitch et al. [7]. This classification characterizes intrahepatic umbilical–portal configurations rather than pathological extrahepatic shunts. In the fetal umbilical–portal venous system, the connection between the MPV and PS is observed in three main morphological configurations. In the T-shaped configuration, the MPV joins the PS in an end-to-side manner, allowing oxygenated blood from the umbilical vein (UV) to be directed into the portal system via the left portal vein (LPV). In the X-shaped configuration, a side-to-side connection exists between the MPV and PS, with the vessels running nearly parallel and forming a characteristic cross-shaped pattern. In the H-shaped configuration, a thin communicating vessel connects the MPV/posterior right portal vein (PRPV) complex with the LPV/anterior right portal vein (ARPV) complex, creating an H-like arrangement (Figure 2).

Anastomosis type was assigned prospectively during the index ultrasound examination. Because fetal biometry, Doppler measurements, and vascular classification were obtained during the same scan, the examiner was aware of the fetal biometry and Doppler findings and was not blinded to the clinical group assignment during image interpretation. No formal repeat classification or intraobserver/interobserver agreement analysis was performed.

Maternal variables included maternal age, pre-pregnancy body mass index, gravida, parity, and nulliparity. Sonographic variables recorded at the index examination included gestational age at assessment, abdominal circumference and percentile, EFW, EFW percentile and Z-score, maximum vertical pocket, umbilical artery systolic/diastolic ratio, UA-PI and percentile, middle cerebral artery pulsatility index (MCA-PI) and percentile, uterine artery pulsatility index and percentile, and CPR and percentile. Obstetric and neonatal variables were obtained from medical records and included gestational age at delivery, preterm birth, fetal sex, birth weight and percentile, cesarean delivery for fetal distress, 1- and 5-min Apgar scores, 5-min Apgar score ≤ 7, NICU admission, length of NICU stay, umbilical artery pH (in NICU cases), transient tachypnea of the newborn (TTN), need for continuous positive airway pressure (CPAP), need for invasive mechanical ventilation, and neonatal sepsis. Clinical and neonatal outcome data were retrieved from the hospital’s electronic medical record system and verified through review of original obstetric and neonatal charts.

The primary neonatal outcome was predefined as composite adverse perinatal outcome (CAPO), defined as the presence of at least one of the following: fetal distress requiring operative delivery, TTN, NICU admission, 5-min Apgar score ≤ 7, need for CPAP, or need for mechanical ventilation. Secondary outcomes included individual CAPO components, as well as gestational age at delivery, birth weight and percentile, length of NICU stay, umbilical artery pH, and neonatal sepsis.

Obstetric management was determined by the attending clinical team according to institutional practice and was independent of the umbilical–portal anastomosis type, which was not used to guide surveillance, timing of delivery, or mode of birth. In pregnancies with late-onset FGR, the decision between continued surveillance and delivery was individualized according to gestational age, the degree of fetal size reduction, amniotic fluid volume, UA/MCA/CPR Doppler findings, and antenatal fetal testing. When delivery was indicated and vaginal birth was not contraindicated, induction of labor was offered. Continuous electronic fetal heart-rate monitoring was used during labor. Cesarean delivery for suspected fetal compromise was performed in the presence of persistent non-reassuring fetal heart rate patterns, pathological cardiotocographic findings, or when immediate delivery was deemed clinically necessary.

### 2.1. Sample Size Calculation

Sample size calculation was performed a priori using G*Power software (version 3.1.9.7; Heinrich Heine University Düsseldorf, Düsseldorf, Germany). The calculation was based on the effect size (w = 0.351) reported by Kaymak and Madazli for differences in the distribution of umbilical–portal anastomosis types between late-onset FGR and AGA fetuses [14]. Assuming a chi-square test of independence, a two-sided significance level of α = 0.05, and 95% statistical power (1 − β = 0.95), the required minimum sample size was calculated as 126 pregnancies. The total sample size of 160 pregnancies in the present study exceeded this requirement and provided adequate statistical power for the primary analysis.

### 2.2. Statistical Analysis

Statistical analysis was performed using IBM SPSS Statistics for Windows, version 27.0 (IBM Corp., Armonk, NY, USA). Continuous variables were assessed for normality using the Shapiro–Wilk test and presented as the mean ± standard deviation or median (interquartile range), as appropriate. Categorical variables were expressed as counts and percentages. Comparisons between groups were performed using the Student’s *t*-test or Mann–Whitney U test for continuous variables and the chi-square or Fisher’s exact test for categorical variables. Multivariable logistic regression analysis was used to evaluate the independent association between umbilical–portal anastomosis type and late-onset FGR, adjusting for potential confounders. Within the FGR cohort, Firth’s penalized logistic regression was applied to assess factors associated with CAPO. aOR with 95% confidence intervals (CI) were reported. A *p*-value < 0.05 was considered statistically significant.

## 3. Results

**Figure 3:**
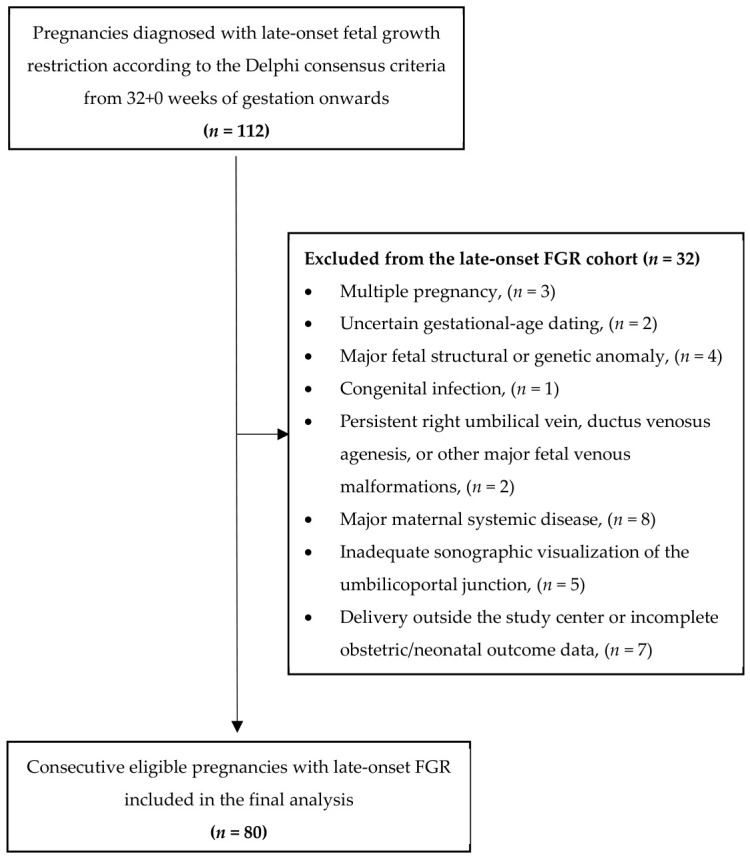
Participant Flow Chart.

During the study period, 112 pregnancies fulfilled the Delphi consensus criteria for late-onset fetal growth restriction. After exclusion of 32 pregnancies according to the predefined eligibility criteria, including major maternal or fetal conditions, inadequate visualization of the umbilicoportal junction, and incomplete outcome data, 80 consecutive pregnancies with late-onset FGR were included in the final analysis (Figure 3). An additional 80 AGA pregnancies meeting the predefined inclusion criteria were consecutively recruited during the same study period as the control group. Accordingly, a total of 160 pregnancies were included: 80 with AGA fetuses and 80 with late-onset FGR.

Baseline maternal and sonographic characteristics are summarized in Table 1. Compared with the AGA group, the late-onset FGR group had a similar maternal age (27 (24–31) vs. 28 (25–33) years, *p* = 0.246) and pre-pregnancy BMI (27.72 (23.85–29.68) vs. 27.51 (23.91–30.47) kg/m^2^, *p* = 0.453), but a significantly higher gestational age at examination (35.2 (33.3–36.3) vs. 33.6 (32.3–35.0) weeks, *p* < 0.001). As expected, fetal biometry was significantly reduced in the late-onset FGR group, including abdominal circumference, abdominal circumference percentile, EFW, EFW percentile, and EFW Z-score (all *p* < 0.001). In addition, pregnancies with late-onset FGR had lower maximum vertical pocket, lower MCA-PI, lower MCA-PI percentile, lower CPR, and lower CPR percentile, together with higher UA-PI and UA-PI percentile (all *p* < 0.001). By contrast, UA systolic/diastolic ratio, uterine artery pulsatility index (UtA-PI), and UtA-PI percentile did not differ significantly between the groups.

The distribution of intrahepatic umbilical–portal venous anastomosis types differed significantly between AGA and late-onset FGR pregnancies (*p* < 0.001). T-shaped anastomosis was more frequent in the AGA group than in the late-onset FGR group (61.3% vs. 26.3%), whereas X-shaped anastomosis was markedly more common in the late-onset FGR group (55.0% vs. 23.8%). H-shaped anastomosis showed a comparable distribution between the two groups (15.0% vs. 18.8%).

Obstetric and neonatal outcomes are presented in Table 2. Pregnancies with late-onset FGR delivered earlier than AGA pregnancies (37.0 (37.0–37.2) vs. 38.5 (37.6–39.2) weeks, *p* < 0.001) and had significantly lower birth weight (2285 (2120–2405) vs. 3165 (2885–3465) g, *p* < 0.001) and birth-weight percentile (2 (1–3) vs. 44 (23–71), *p* < 0.001). Among the 80 neonates in the late-onset FGR group, 78 (97.5%) had a birth weight below the 10th percentile. Neonates in the late-onset FGR group also had lower 1-min and 5-min Apgar scores (both *p* < 0.001), higher rates of NICU admission (21.3% vs. 6.3%, *p* = 0.006), and a higher incidence of TTN (17.5% vs. 6.3%, *p* = 0.028). The rate of CAPO was also significantly higher in the late-onset FGR group than in the AGA group (35.0% vs. 15.0%, *p* = 0.003). Although preterm birth, cesarean delivery for fetal distress, 5-min Apgar score ≤ 7, and need for CPAP were numerically more frequent in the late-onset FGR group, these differences did not reach statistical significance. NICU length of stay, umbilical artery pH among NICU-admitted neonates, need for mechanical ventilation, and neonatal sepsis were similar between groups.

Within the late-onset FGR cohort, 28 of 80 pregnancies (35.0%) developed CAPO (Table 3). Maternal age, pre-pregnancy body mass index, gravidity, parity, nulliparity rate, gestational age at examination, gestational age at delivery, birth weight, and birth-weight percentile were comparable between the CAPO and non-CAPO subgroups (all *p* > 0.05). Likewise, no statistically significant differences were observed in fetal biometry, amniotic fluid volume, UA S/D ratio, UA-PI, MCA-PI, UtA-PI, or CPR and their corresponding percentiles. Although the overall distribution of anastomosis type did not reach statistical significance on univariable comparison (*p* = 0.085), X-shaped anastomosis was more frequent among pregnancies with CAPO than among those without CAPO (71.4% vs. 46.2%), whereas T-shaped anastomosis was less frequent (14.3% vs. 32.7%).

In multivariable analysis (Table 4), with T-shaped anastomosis used as the reference category, both X-shaped and H-shaped anastomoses were independently associated with late-onset FGR after adjustment for maternal age and pre-pregnancy body mass index. X-shaped anastomosis showed the strongest association (aOR, 5.269; 95% CI, 2.484–11.177; *p* < 0.001), whereas H-shaped anastomosis was also significantly associated with late-onset FGR (aOR, 2.845; 95% CI, 1.124–7.201; *p* = 0.027). Among pregnancies with late-onset FGR, Firth’s penalized logistic regression adjusted for birth-weight percentile demonstrated that X-shaped anastomosis was independently associated with CAPO (aOR, 3.341; 95% CI, 1.003–11.133; *p* = 0.049), while H-shaped anastomosis was not significantly associated with CAPO (aOR, 1.513; 95% CI, 0.337–6.790; *p* = 0.589).

Consistent with the multivariable findings, adjusted predicted probabilities showed that the probability of late-onset FGR was highest in pregnancies with X-shaped anastomosis (0.696; 95% CI, 0.581–0.810), intermediate in those with H-shaped anastomosis (0.553; 95% CI, 0.364–0.741), and lowest in those with T-shaped anastomosis (0.303; 95% CI, 0.194–0.413) (Table 5). Pairwise comparisons demonstrated a significantly higher predicted probability of late-onset FGR for X-shaped versus T-shaped anastomosis (absolute difference, 0.392; 95% CI, 0.233–0.552; *p* < 0.001) and for H-shaped versus T-shaped anastomosis (absolute difference, 0.249; 95% CI, 0.030–0.469; *p* = 0.026). By contrast, the difference between H-shaped and X-shaped anastomosis was not statistically significant (absolute difference, −0.143; 95% CI, −0.363 to 0.077; *p* = 0.202).

## 4. Discussion

In this prospective case–control study, we found that the distribution of fetal intrahepatic umbilical–portal anastomosis types differed significantly between late-onset FGR and AGA pregnancies, with a marked overrepresentation of the X-shaped pattern in the FGR group and a relative predominance of the T-shaped pattern in controls. Importantly, X-shaped anastomosis was independently associated not only with late-onset FGR but also with CAPO within the FGR cohort. In addition, H-shaped anastomosis was also independently associated with late-onset FGR, although its association with adverse perinatal outcome was not observed. While conventional Doppler indices clearly distinguished late-onset FGR from AGA fetuses, they did not discriminate CAPO within the FGR group, whereas anastomosis type did. These findings suggest that umbilical–portal anatomy may capture clinically relevant information not fully reflected by conventional arterial Doppler at a single examination.

The late-onset FGR group underwent ultrasound examination at a more advanced gestational age than the AGA group, most likely reflecting the timing of referral and diagnosis in a late-onset condition. The anastomosis categories describe vascular geometry rather than a gestation-dependent quantitative measurement, and only examinations with adequate visualization of the umbilicoportal junction were included; therefore, gestational age alone is unlikely to explain the marked shift in configuration. Nevertheless, advancing gestation may affect the ease of visualizing small intrahepatic vessels and is directly relevant to the interpretation of Doppler indices. Because gestational age at examination was not included in the primary multivariable model, residual confounding or differential visualization cannot be excluded. Future studies should use gestational-age-matched groups and report sensitivity analyses adjusted for gestational age.

Unlike early-onset disease, late-onset FGR is characterized by relatively subtle placental lesions and modest Doppler abnormalities, with a narrow clinical window between apparent stability and neonatal compromise. In this context, markers reflecting fetal adaptive physiology are particularly valuable. Our findings are consistent with those of Kaymak and Madazli, who reported a higher prevalence of X-shaped anastomosis in late-onset FGR and its association with short-term neonatal morbidity [14]. In our study, the distribution of anastomosis types in AGA fetuses was broadly consistent with previously reported normal patterns [6,7], whereas late-onset FGR showed a clear shift toward X-shaped predominance with a relative reduction in T-shaped configuration and stable H-shaped proportions. Within this spectrum, while X-shaped anastomosis appears to represent the most clinically relevant configuration, the association of H-shaped patterns with FGR further supports the concept that variations in umbilical–portal configuration as a whole may carry pathophysiological significance. The associations of the X-shaped and H-shaped configurations with FGR persisted after multivariable adjustment, whereas only the X-shaped configuration was associated with CAPO within the FGR subgroup. Together, these findings support the concept that anastomotic configuration may represent an independent marker of fetal vulnerability.

Our conventional ultrasound findings were largely consistent with the established hemodynamic profile of late-onset FGR. Compared with AGA fetuses, the FGR group had higher UA-PI and lower MCA-PI and CPR, in line with prospective evidence showing that cerebroplacental redistribution and placental resistance remain central components of late-onset disease [15,16]. Rizzo et al. showed that, at the time of diagnosis, lower CPR and reduced umbilical venous flow normalized for abdominal circumference were associated with adverse perinatal outcome in late-onset FGR, underscoring that both arterial redistribution and venous supply are relevant to clinical deterioration [15]. Hamidi et al. similarly reported reduced umbilical venous volume flow in late-onset FGR, even when traditional arterial markers were not uniformly abnormal, suggesting that venous compromise may precede or complement standard Doppler deterioration [12]. In contrast, within our FGR cohort, CAPO and non-CAPO pregnancies did not differ significantly in routine arterial Doppler indices. We think this may reflect the fact that our cohort was restricted to late-onset cases with relatively homogeneous third-trimester surveillance, in whom conventional Doppler variables may cluster within a limited range, while anastomotic configuration may capture a more constitutive difference in hepatic perfusion efficiency under stress.

This interpretation becomes more compelling when our data are placed alongside studies of the fetal venous liver circulation. Kessler et al. demonstrated that growth-restricted fetuses preferentially direct umbilical blood flow to the left hepatic lobe at the expense of the right lobe, indicating that hepatic perfusion is actively redistributed in response to placental insufficiency [10]. Ebbing et al. further showed that FGR is associated with a characteristic redistribution pattern of liver circulation involving altered contributions from the umbilical and portal venous systems, reinforcing the concept that the fetal liver is not a passive recipient of flow but a dynamic participant in adaptation [11]. More recently, Kivilevitch et al. observed that fetuses born small for gestational age exhibit progressive changes in umbilical and portal venous blood-flow volumes across gestation, with evidence of additional redistribution of liver perfusion compared with normally grown fetuses [17]. Taken together, these studies suggest that when placental inflow decreases, the configuration of the umbilicoportal junction may become functionally important by determining how a limited amount of oxygenated blood is distributed within the hepatic–ductus venosus unit.

Our findings should be interpreted within a distinct pathophysiological framework from that of the umbilical–portal–systemic venous shunt literature. Czeiger et al. reported that overt venous shunts are not uncommon in growth-restricted fetuses and are associated with earlier diagnosis, earlier delivery, and poorer perinatal outcomes [18]. In contrast, in the present study, major venous malformations, including persistent right umbilical vein and ductus venosus agenesis, were deliberately excluded. Accordingly, our analysis focused on intrahepatic umbilical–portal anastomotic configurations that appear structurally normal. This distinction is important, as clinically meaningful variation in late-onset FGR may exist even in the absence of overt vascular anomalies. In other words, the key issue may not be complete hepatic bypass, but rather subtle differences in how reduced placental inflow is distributed within the liver after reaching the portal sinus.

These observations are further supported by emerging experimental and clinical evidence on hepatic adaptation and fetal growth signaling. Recent studies of fetal liver circulation have renewed attention to the hepatic arterial buffer response, whereby a reduction in portal flow is compensated by an increase in hepatic arterial flow, contributing to the maintenance of hepatic perfusion [19,20]. In late-onset FGR, abnormalities in hepatic artery Doppler have been associated with both the diagnosis of FGR and adverse neonatal outcomes [19]. Beyond hemodynamic regulation, the fetal liver plays a central role in the insulin-like growth factor (IGF) axis. Placental mTOR signaling has been shown to regulate IGFBP-1 phosphorylation and IGF-1 bioavailability within the fetal liver [21]. In addition, umbilical cord IGF-1 levels have been associated with fetal soft tissue growth and birth weight percentile in late gestation [22]. Taken together, these findings suggest that differences in flow distribution at the umbilicoportal junction may influence not only oxygen delivery but also liver-mediated growth signaling pathways.

The clinical implication of our findings is not that anastomosis type should replace established Doppler surveillance, but that it may serve as a complementary marker in late-onset FGR, particularly when routine arterial findings are borderline or do not fully explain neonatal outcomes. This is especially relevant in clinical situations where decision-making requires balancing modest sonographic abnormalities against the risk of unexpected fetal compromise. Studies on CPR have shown an association with emergency cesarean delivery for fetal distress, but only moderate predictive performance, highlighting the limitations of relying on a single functional index [23]. In this context, a simple anatomical feature that can be readily assessed during standard upper abdominal examination may enhance current risk stratification. If validated in larger prospective studies, the presence of an X-shaped anastomosis may help identify late-onset FGR pregnancies that could benefit from closer surveillance or additional venous and hepatic Doppler assessment.

Because CAPO included fetal distress requiring operative delivery, one component of the composite outcome is inherently influenced by obstetric management. Although anastomosis type was not used to guide clinical decision-making and all participants were managed according to the same institutional protocol, differences in induction thresholds, interpretation of intrapartum cardiotocography, and clinician judgment may have affected the operative-delivery rate. Therefore, the observed associations between umbilical–portal anastomosis type and adverse perinatal outcomes should be interpreted in the context of local obstetric practice and confirmed in multicenter studies using standardized surveillance and delivery protocols.

A major strength of this study is its focus on intrahepatic umbilical–portal anastomotic variations that do not constitute overt venous malformations but have largely been overlooked in late-onset FGR. The finding that anastomosis type is associated not only with late-onset FGR but also with CAPO within the same cohort suggests that these structural variations may have clinical relevance beyond a purely anatomical observation. In particular, in situations where arterial Doppler parameters fail to discriminate adverse outcomes within the FGR group, the additional information provided by anastomosis type represents a notable strength of the study. This study has several limitations. Its single-center design and modest sample size, particularly the low number of adverse events in the late-onset FGR subgroup, may limit generalizability. All ultrasound examinations and anastomosis classifications were performed by a single experienced examiner. Although this approach enhanced procedural consistency, it introduces operator dependence and limits external reproducibility. The absence of formal intraobserver and interobserver agreement analyses prevents quantification of classification reliability and may reduce the generalizability of the findings. In addition, the analysis was based on morphological classification without direct physiological measurements to confirm functional implications. Therefore, the proposed mechanism remains inferential and requires further validation. Finally, outcomes were limited to short-term perinatal events, and long-term implications remain unknown.

## 5. Conclusions

In this prospective case–control study, intrahepatic umbilical–portal anastomotic configuration was independently associated with late-onset FGR, with the strongest association observed for the X-shaped pattern and a lesser but significant association for the H-shaped pattern. Adjusted predicted probabilities demonstrated a clear gradient in FGR risk across anastomosis types, increasing from T-shaped to H-shaped and reaching the highest levels in X-shaped configurations. Within the late-onset FGR cohort, only X-shaped anastomosis remained independently associated with CAPO. Together, these findings suggest that intrahepatic umbilical–portal vascular configuration, particularly the X-shaped pattern, captures clinically relevant information beyond conventional fetal biometry and arterial Doppler. Given that this parameter can be readily assessed during routine ultrasound examination, it may serve as a complementary marker for risk stratification, although further multicenter studies integrating functional and long-term outcome data are required before clinical implementation.

## Figures and Tables

**Table 1:** Clinical characteristics of the appropriate-for-gestational-age and late-onset fetal growth restriction groups.

Variables	Appropriate-for-Gestational-Age	Late-Onset Fetal Growth Restriction	*p*-Value
Demographic Parameters			
Maternal age (years)	28 (25–33)	27 (24–31)	0.246 ^a^
Pre-pregnancy body mass index (kg/m^2^)	27.51 (23.91–30.47)	27.72 (23.85–29.68)	0.453 ^a^
Gravidity	2 (1–3)	2 (1–3)	0.814 ^a^
Parity	1 (0–1)	1 (0–2)	0.841 ^a^
Nulliparity	34 (42.5%)	37 (46.3%)	0.633 ^a^
GA at examination (weeks)	33.6 (32.3–35.0)	35.2 (33.3–36.3)	<0.001 ^a^
Ultrasonographic Parameters			
AC measurement (mm)	298.9 (285.6–310.7)	274.9 (258.7–290.2)	<0.001 ^a^
AC measurement percentile	50.3 (30.0–73.4)	1.0 (1.0–2.1)	<0.001 ^a^
EFW (gram)	2307 ± 349	1943 ± 360	<0.001 ^b^
EFW percentile	45.0 (26.5–62.5)	3.0 (2.0–6.0)	<0.001 ^a^
EFW Z-score	−0.11 (−0.63 to 0.31)	−1.88 (−2.11 to −1.58)	<0.001 ^a^
MVP (mm)	55 (50–62)	50 (40–58)	<0.001 ^a^
UA S/D	2.40 (2.20–2.60)	2.50 (2.20–2.92)	0.067 ^a^
UA-PI	0.76 (0.70–0.84)	0.90 (0.78–1.01)	<0.001 ^a^
UA-PI percentile	8.63 (3.88–19.29)	46.61 (12.37–70.90)	<0.001 ^a^
MCA-PI	1.86 (1.76–1.96)	1.44 (1.35–1.51)	<0.001 ^a^
MCA-PI Percentile	47.10 (41.52–59.61)	6.99 (6.10–8.81)	<0.001 ^a^
UtA-PI	0.74 (0.69–0.77)	0.67 (0.61–0.83)	0.054 ^a^
UtA-PI Percentile	52.33 (47.99–62.78)	46.77 (26.50–74.92)	0.194 ^a^
CPR	2.43 (2.21–2.67)	1.59 (1.41–1.86)	<0.001 ^a^
CPR Percentile	84.88 (70.28–92.95)	15.50 (5.70–41.25)	<0.001 ^a^
Umbilical–portal anastomosis type			<0.001 ^c^
T-shaped	49 (61.3%)	21 (26.3%)	
X-shaped	19 (23.8%)	44 (55.0%)	
H-shaped	12 (15.0%)	15 (18.8%)	

^a^ The Mann–Whitney U test was used for comparisons between groups. Data are presented as median (interquartile range). ^b^ Independent-samples *t*-test was used for comparisons between groups. Data are presented as the mean ± standard deviation. ^c^ Categorical variables were compared using the chi-square or Fisher’s exact test, as appropriate. Results are shown as *n* (%). Abbreviations: GA: Gestational age; AC: Abdominal circumference; EFW: Estimated fetal weight; MVP: Maximum vertical pocket; UA: Umbilical artery; S/D: Systolic/diastolic ratio; PI: Pulsatility index; MCA: Middle cerebral artery; UtA: Uterine artery; CPR: Cerebroplacental ratio.

**Table 2:** Obstetric and perinatal outcomes in the appropriate-for-gestational-age and late-onset fetal growth restriction groups.

Variables	Appropriate-for-Gestational-Age	Late-Onset Fetal Growth Restriction	*p*-Value
Gestational age at delivery (weeks)	38.5 (37.6–39.2)	37.0 (37.0–37.2)	<0.001 ^a^
Preterm birth	6 (7.5%)	12 (15.0%)	0.133 ^a^
Fetal Sex			0.752 ^c^
Male	39 (48.8%)	37 (46.3%)	
Female	41 (51.2%)	43 (53.8%)	
Birth weight (gram)	3165 (2885–3465)	2285 (2120–2405)	<0.001 ^a^
Birth weight percentile	44 (23–71)	2 (1–3)	<0.001 ^a^
C/S fetal distress	9 (11.3%)	16 (20.0%)	0.127 ^c^
1-min Apgar score	9 (9–9)	8 (8–9)	<0.001 ^a^
5-min Apgar score	10 (10–10)	9 (9–10)	<0.001 ^a^
5-min Apgar score ≤ 7	1 (1.3%)	6 (7.5%)	0.117 ^c^
NICU admission	5 (6.3%)	17 (21.3%)	0.006 ^c^
NICU length of stay (days)	7 (7–9)	8 (6–11)	0.844 ^a^
Umbilical artery pH	7.30 ± 0.10	7.29 ± 0.08	0.760 ^b^
TTN	5 (6.3%)	14 (17.5%)	0.028 ^c^
Need for CPAP	5 (6.3%)	11 (13.8%)	0.114 ^c^
Need for mechanical ventilation	1 (1.3%)	1 (1.3%)	>0.99 ^c^
Sepsis	-	1 (1.3%)	>0.99 ^c^
CAPO	12 (15.0%)	28 (35.0%)	0.003 ^c^

^a^ The Mann–Whitney U test was used for comparisons between groups. Data are presented as median (interquartile range). ^b^ Independent-samples *t*-test was used for comparisons between groups. Data are presented as the mean ± standard deviation. ^c^ Categorical variables were compared using the chi-square or Fisher’s exact test, as appropriate. Results are shown as *n* (%). Abbreviations: C/S: Cesarean section; NICU: Neonatal intensive care unit; TTN: Transient tachypnea of the newborn; CPAP: Continuous positive airway pressure; CAPO: Composite adverse perinatal outcome.

**Table 3:** Maternal, obstetric, sonographic, and neonatal characteristics according to CAPO status in pregnancies with late-onset FGR.

Variables	CAPO (*n* = 28)	Non-CAPO (*n* = 52)	*p*-Value
Demographic Parameters			
Maternal age (years)	27 (24–31)	28 (24–31)	0.907 ^a^
Pre-pregnancy body mass index (kg/m^2^)	27.92 (25.06–29.91)	27.37 (22.88–39.43)	0.254 ^a^
Gravidity	2 (1–2)	2 (1–3)	0.165 ^a^
Parity	0 (0–1)	1 (0–2)	0.184 ^a^
Nulliparity	16 (57.1%)	21 (40.4%)	0.152 ^a^
GA at examination (weeks)	34.5 (33.2–36.5)	35.3 (33.4–36.3)	0.610 ^a^
Obstetric Characteristics			
Gestational age at delivery (weeks)	37.1 (37.0–37.3)	37.0 (37.0–37.2)	0.958 ^a^
Birth weight (gram)	2235 (2075–2415)	2300 (2150–2385)	0.586 ^a^
Birth weight percentile	1 (1–3)	2 (1–3)	0.481 ^a^
Ultrasonographic Parameters			
AC measurement (mm)	270 (259–286)	279 (258–292)	0.426 ^a^
AC measurement percentile	1.0 (1.0–2.1)	1.1 (1.0–2.1)	0.716 ^a^
EFW (gram)	1901 ± 348	1966 ± 367	0.445 ^b^
EFW percentile	3.0 (1.5–5.5)	4.0 (2.0–6.0)	0.390 ^a^
EFW Z-score	−1.91 (−2.19 to −1.57)	−1.81 (−2.08 to −1.58)	0.449 ^a^
MVP (mm)	48 (34–55)	50 (41–60)	0.218 ^a^
UA S/D	2.46 (2.20–2.98)	2.58 (2.24–2.92)	0.774 ^a^
UA-PI	0.91 ± 0.18	0.91 ± 0.21	0.962 ^b^
UA-PI percentile	39.09 (14.53–69.00)	50.97 (12.00–73.60)	0.832 ^a^
MCA-PI	1.46 (1.33–1.51)	1.41 (1.35–1.50)	0.650 ^a^
MCA-PI Percentile	6.86 (5.56–8.89)	7.19 (6.21–8.79)	0.675 ^a^
UtA-PI	0.67 (0.60–0.79)	0.68 (0.62–0.83)	0.720 ^a^
UtA-PI Percentile	39.60 (25.52–73.43)	48.35 (27.85–76.22)	0.635 ^a^
CPR	1.63 ± 0.29	1.64 ± 0.40	0.875 ^b^
CPR Percentile	18.73 (6.57–40.37)	14.73 (4.81–41.98)	0.952 ^a^
Umbilical–portal anastomosis type			0.085 ^c^
T-shaped	4 (14.3%)	17 (32.7%)	
X-shaped	20 (71.4%)	24 (46.2%)	
H-shaped	4 (14.3%)	11 (21.2%)	

^a^ The Mann–Whitney U test was used for comparisons between groups. Data are presented as median (interquartile range). ^b^ Independent-samples *t*-test was used for comparisons between groups. Data are presented as the mean ± standard deviation. ^c^ Categorical variables were compared using the chi-square or Fisher’s exact test, as appropriate. Results are shown as *n* (%). Abbreviations: GA: Gestational age; AC: Abdominal circumference; EFW: Estimated fetal weight; MVP: Maximum vertical pocket; UA: Umbilical artery; S/D: Systolic/diastolic ratio; PI: Pulsatility index; MCA: Middle cerebral artery; UtA: Uterine artery; CPR: Cerebroplacental ratio; CAPO: Composite adverse perinatal outcome.

**Table 4:** Association of intrahepatic umbilical–portal venous anastomosis type with late-onset FGR and CAPO.

Outcome/Model	Predictor (Reference: T-Shaped)	aOR	95% CI	*p*-Value
Late-onset FGR	X-shaped	5.269	2.484–11.177	<0.001
	H-shaped	2.845	1.124–7.201	0.027
CAPO among LO-FGR cohort (Firth model)	X-shaped	3.341	1.003–11.133	0.049
	H-shaped	1.513	0.337–6.790	0.589

Abbreviations: aOR indicates adjusted odds ratio; CI, confidence interval; CAPO, composite adverse perinatal outcome; LO-FGR, late-onset fetal growth restriction; BMI, body mass index. Note: The LO-FGR model was adjusted for maternal age and pre-pregnancy BMI. The CAPO model within the LO-FGR cohort was estimated using Firth’s penalized logistic regression (to reduce small-sample bias and potential separation) and was adjusted for birth weight percentile.

**Table 5:** Adjusted predicted probabilities of FGR by intrahepatic umbilical–portal venous anastomosis type and pairwise comparisons.

**Anastomosis Type**	**Predicted Probability of FGR**	**95% CI**	***p*-Value**
T-shaped	0.303	0.194–0.413	<0.001
X-shaped	0.696	0.581–0.810	<0.001
H-shaped	0.553	0.364–0.741	<0.001
**Pairwise Comparisons of Adjusted Predicted Probabilities**			
**Comparison**	**Absolute Difference in Predicted Probability**	**95% CI**	***p*-Value**
X-shaped vs. T-shaped	0.392	0.233–0.552	<0.001
H-shaped vs. T-shaped	0.249	0.030–0.469	0.026
H-shaped vs. X-shaped	−0.143	−0.363–0.077	0.202

Note: Adjusted predicted probabilities were derived from a logistic regression model using the margins command (delta method). Differences represent absolute risk differences between anastomosis types. CI: confidence interval.

## Data Availability

The datasets generated and analyzed during the current study are not publicly available due to institutional data protection policies, but are available from the corresponding author on reasonable request.
